# The association between meeting physical activity guidelines and academic performance among junior high school adolescents in China-evidence from the China education tracking survey

**DOI:** 10.3389/fpsyg.2023.1002839

**Published:** 2023-02-16

**Authors:** Jie Yang, Hai Wang, Lin Luo

**Affiliations:** ^1^College of Exercise and Health, Shandong Sport University, Rizhao, China; ^2^Guizhou Provincial Academy of Education, Guiyang, China; ^3^College of Physical Education, Guizhou Normal University, Guiyang, China; ^4^Basic Education Research Center, Southwest University, Chongqing, China

**Keywords:** physical activity guidelines, physical exercise time, screen time, academic performance, adolescent

## Abstract

**Purpose:**

To examine how well Chinese adolescents meet the physical exercise time and screen time recommendations in the Physical Activity Guidelines for Chinese Residents (2021), and the relationship between adolescents’ physical exercise time, screen time and their academic performance.

**Method:**

Daily physical exercise time, screen time and academic performance were collected from Grade 8 adolescents (*n* = 9,449). Academic performance included standardized scores on Chinese, Math and English tests and responses to the School Life Experience Scale.

**Results:**

Meeting the physical activity time and screen time in the Physical Activity Guidelines for Chinese Residents was associated with adolescents’ academic performance. Specifically, having at least 60 min of physical exercise per day was associated with adolescents’ school life experiences compared to adolescents who did not meet the physical exercise time and screen time in the Chinese Residents’ Physical Activity Guidelines. Less than 2  h of cumulative screen time per day was associated with adolescent mathematics test scores, English test scores and school life experiences. Meeting both physical exercise time and screen time to be recommended had more significant effects on adolescents’ mathematics, Chinese, English and school life experiences. Meeting both the physical exercise time and screen time recommendations in the Physical Activity Guidelines for Chinese Residents was more significantly associated with boys’ mathematics test scores, Chinese test scores and School life experience. Meeting both the physical exercise time and screen time requirements in the Physical Activity Guidelines for Chinese Residents had a more significant effect on School life experience for girls.

**Conclusion:**

Physical exercise participation of at least 60 min per day and/or less than 2  h of cumulative screen time per day were both associated with adolescent academic performance. Stakeholders should actively promote adolescents to follow the Physical Activity Guidelines for Chinese Residents (2021).

## Introduction

Previous studies have extensively examined the relationship between adequate physical exercise, limiting screen time and the physical and mental health of children and adolescents (Keane et al., 2017; Rodriguez-Ayllon et al., 2019; Xiao et al., 2021). Increasing regular physical activity may help to improve adolescent physical health and reduce obesity (Verstraete et al., 2007; Augestad and Jiang, 2015). High levels of physical activity have been shown to be associated with mental health, including depression, anxiety and stress in children and adolescents (Xiao et al., 2021). Physical exercise can also alleviate negative emotions in adolescents (Ma et al., 2020). To maintain optimal physical and mental health, the World Health Organization recommended that children and adolescents aged 5–17 years should engage in at least 60 min of moderate to vigorous physical activity each day (World Health Organization, 2018). Decreased physical activity is associated with increased screen time in children and adolescents (Hands et al., 2011). This in turn increases the risk of obesity, negative mood and other behavioral problems in adolescents (Twenge et al., 2018).

There has been some research evidence that physical exercise and screen time are also associated with academic performance in children and adolescents. For example, the findings of Alvarez-Bueno et al. (2017) showed that physical activity, especially physical exercise improved classroom behavior and benefited several aspects of academic achievement, particularly math-related skills, reading and overall scores of adolescents (Alvarez-Bueno et al., 2017). García-Hermoso et al. (2021) reported cognitive performance and academic achievement had significant improvements in children and adolescents through a physical exercise intervention in 8,676 adolescents aged 5–18 years (García-Hermoso et al., 2021). Adelantado-Renau et al. (2019) conducted a study of 480,479 children and adolescents aged 4–18 years. Their study showed that watching television and playing video games (not all screen media) were negatively associated with academic performance in children and adolescents, and appeared to have a greater impact on adolescents than children (Adelantado-Renau et al., 2019). Zhang et al. (2022) conducted a study on the relationship between screen time and health in Chinese school-age children and adolescents, which found that 2 h of screen time per day was associated with poorer academic performance in adolescents (Zhang et al., 2022). Thus, children and adolescents with adequate physical exercise time and less screen time are likely to perform better academically. However, these behavioral studies have mainly examined these two elements in isolation, with only a few studies examining their combined effects on children’s and adolescents’ academic performance. For example, Marciano and Camerini (2021) conducted a study of 1,208 adolescents with a mean age of 13.55 years and their study reported that adequate physical exercise (at least 60 min/day) and limited screen time (<2 h/day) were associated with higher academic achievement (Marciano and Camerini, 2021).

The 2021 Physical Activity Guidelines for the Chinese Population proposes the integration of physical exercise and screen time as key components of the physical activity guidelines for children and adolescents aged 6–17 years, due to their interactions and interdependence. The physical activity guidelines suggest that children and adolescents aged 6–17 years should have at least 60 min of physical exercise per day and less than 2 h of screen time per day in aggregate. Although several previous studies have examined the effectiveness of physical exercise and screen time in predicting adolescent health outcomes, there is still a lack of sufficient evidence to support the relationship between meeting physical activity guidelines for Chinese populations and the academic performance of Chinese adolescents. There is therefore a need to conduct studies in Chinese adolescent samples to further confirm the relationship between meeting the Physical Activity Guidelines for Chinese Populations and the academic performance of Chinese adolescents.

## Materials and methods

### Research design and participants

This study analyzed primary data from the China Education Tracking Survey (CEPS).

The CEPS is a national cohort study designed to document the development and attributes of the educational experiences of Chinese adolescents. The CEPS study adopted a multi-stage stratified design. The CEPS stratified counties (or equivalent administrative areas), schools and classes into primary, secondary and tertiary sampling units. Primary sampling units were stratified by region and migrant population size, with counties in Shanghai or other areas with high migrant populations being oversampled. Four schools were sampled from each of the sampled counties using a probability proportional to size method. Two classrooms were sampled from each school. Baseline data was drawn from a total of 28 cities or counties in China, including 112 schools and 224 classes. All students in the sample classrooms were asked to complete a self-administered questionnaire administered by a local survey team composed of trained researchers from a provincial university or provincial social science college.

This study focused on the analysis of adolescent follow-up data from grade 8 from 2014 to 2015.The content involved data on adolescent and parent demographic characteristics, physical activity status, and academic performance. The CEPS 2014–2015 follow-up data sample size was 9,920 (follow-up sample attrition rate of 7.72%, 830; new sample of 471). Non-missing data for all variables of interest were included in the final data analysis. The study analyzed information from only 9,449 (4,842 males, 4,436 females, 171 not reported by sex) participants (13–17 years old, mean age 14.65 ± 0.01 years) for whom complete data were available. The study was approved by the ethics committee of Renmin University of China. The children signed consent forms and their parents signed consent forms to participate in this study. Students were not rewarded for their participation.

## Key variables and measurement methods

### Outcome variables

Two types of academic performance indicators were used in this study: standardized subject test scores and scores on the School Life Experience Scale (Ma et al., 2020).

Standardized subject test scores will be included in the final exam results for Math, Language Arts, and English in grade K8. These scores are extracted from the school’s student records by a specially trained CEPS team (Duan et al., 2018). These three subjects are considered the basic core curriculum of the current Chinese education system. In this study, the raw scores of each subject score were first standardized by school and converted to a Z-score with a mean of 0 and a standard deviation of 1, which was then converted to a test score variable taking values from 0 to 100 (Lei and Lee, 2021). This is the most common method used to standardize test scores in the Chinese education system. These standardized subject test scores are comparable across adolescents in the same school year.

Most of the existing studies focus on adolescent academic performance in terms of subject scores, but adolescents’ school-related experiences and attitudes can be considered as important indicators of academic performance (Mok and Flynn, 1997). For example, more positive “school life experiences” contribute significantly to adolescents’ academic performance (Fong Lam et al., 2015). School life experience scores were measured by a 10-item Chinese scale created by the CEPS team. Students were asked to indicate their level of agreement with a 4-point likert scale ranging from “strongly disagree” = 1 to “strongly agree” = 4. Test scores reflect students’ experiences and attitudes toward school life, including general feelings about school life (e.g., I feel good about this school). For example, I am bored with this school) as well as perceptions of specific aspects of school. Scores for each item and the total score were calculated by aggregating all items (four of which were reverse scored). Scores ranged from 10 to 40 points. Higher scores represent students with more positive perceptions and energy about school life. The Cronbach alpha for this scale = 0.72.

### Exposure variables

Meeting physical activity guidelines recommended type. This study focused on adolescents’ achievement of physical exercise and screen time recommendations in physical activity guidelines.

The CEPS used the questions “How many times a week do you usually do physical exercise” and “How many minutes do you do physical exercise each time” to measure the physical exercise status of adolescents. The answers to these two questions were used to obtain the daily physical exercise time of adolescents.

The CEPS uses the question “From Monday to Friday, how much time do you usually spend watching TV each day?” “From Monday to Friday, how much time do you usually spend online and playing games each day?” “On weekends, how much time do you usually spend watching TV each day?” “On weekends, how much time do you usually spend each day surfing the Internet and playing games?” Four questions were used to measure adolescents’ screen time. In this study, the answers to these four questions were used to obtain the daily screen time achievement of adolescents.

Based on the findings of the CEPS, this study classified adolescents’ meeting of physical exercise time and screen time as recommended by the Physical Activity Guidelines for Chinese People into four categories. In the first type, both physical exercise time and screen time did not meet the guidelines (NPA + NST); in the second type, only physical exercise time was meeting the guidelines (PA + NST); in the third type, only screen time was meeting the guidelines (NPA + ST); and in the fourth type, both physical exercise time and screen time were meeting the guidelines (PA + ST).

### Covariates

Covariates included students’ demographic, physical and family characteristics. These included age (years), gender, race (Han, non-Han), only child (yes, no), household registration (urban, rural), parental education level (≤ middle school = 1, high school or vocational school = 2, ≥ college = 3) and perceived household socioeconomic status (low income = 1, middle class = 2, rich = 3).

Body size was measured using body mass index (BMI) as a proxy. BMI, calculated by dividing the student’s self-reported weight by the square of self-reported height (Ma et al., 2020). This study used the “WS/T 586–2018 Screening for Overweight and Obesity in School-Aged Children and Adolescents” standard, which is a set of Chinese national standards recommended by the National Health Council of China for adolescents aged 6 to 18 years (WS/T 586–2018) (National Health Commission, 2018).

## Statistical analysis

Categorical variables are described using frequency percentages. Continuous variables were described using the mean (standard deviation). Comparisons of categorical variables between genders were made using chi-square tests and continuous variables using Kruskal–Wallis test. The Kruskal–Wallis test was used to analyze between-group differences in adolescent academic performance. Logistic regression was used to analyze the relationship between different types of physical exercise time and types of screen time combinations and adolescent academic performance. The study also looked at gender stratification differences in the study results. The Stata 16 software (StataCorp LP, College Station, Texas) was used for data analysis. Statistical significance was set at *p* < 0.05.

## Results

### Characteristics of adolescents

The characteristics of the adolescents in the study sample are shown in Table 1. Overall, 3.81% of the 9,449 participants meeting the physical exercise time required by the Physical Activity Guidelines for Chinese Populations, 17.62% meeting the screen time required by the Physical Activity Guidelines for Chinese Populations, and 1.01% meeting both the physical exercise time and screen time requirements of the Physical Activity Guidelines for Chinese Populations.

**Table 1:** Characteristics of Chinese adolescents [mean (SD)/%].

	All (*n* = 9,449)	Boys (*n* = 4,842)	Girls (*n* = 4,436)	*X* ^2^	P for sex difference
Age(y)	14.57 (0.01)	14.65 (0.01)	14.49 (0.10)	98.924	**<0.001**
Ethnicity				1.049	0.306
Han	90.88	91.16	90.57		
Non-Han	9.12	8.84	9.43		
Residence				5.432	**0.020**
City	53.82	54.89	52.59		
Rural	46.18	45.11	47.41		
Single child				21.481	**<0.001**
Yes	57.03	54.92	59.46		
No	42.97	45.08	40.54		
Father’s highest education				10.039	**0.007**
≤ Junior middle school	57.70	58.90	56.32		
Senior middle school or vocational school	26.47	26.23	26.75		
≥ College	15.83	14.87	16.93		
Mother’s highest education				4.295	0.117
≤ Junior middle school	64.23	64.73	63.65		
Senior middle school or vocational school	22.54	22.68	23.38		
≥ College	13.23	12.59	13.97		
Perceived household economic status				18.801	**<0.001**
Lower income	21.50	22.83	19.97		
Middle class	72.63	70.86	74.67		
Wealthy	5.87	6.31	5.35		
BMI(kg/m^2^)				92.501	**<0.001**
Slimmer	44.20	42.99	45.59		
Normal	37.06	35.02	39.41		
Overweight	5.18	6.59	3.54		
Obesity	13.57	15.40	11.46		
Meeting the guide categories				214.484	**<0.001**
NPA + NST	77.56	78.93	75.97		
PA + NST	3.81	5.76	1.57		
NPA + ST	17.62	41.04	21.75		
PA + ST	1.01	1.07	0.71		
Chinese test scores	50.00 (0.10)	47.56 (0.15)	52.76 (0.12)	646.826	**<0.001**
Mathematics test scores	50.00 (0.10)	48.88 (0.14)	51.26 (0.14)	110.809	**<0.001**
English test scores	50.00 (0.10)	47.53 (0.14)	52.78 (0.13)	623.880	**<0.001**
School life experience	23.82 (0.04)	23.98 (0.06)	23.65 (0.06)	23.132	**<0.001**

*p*-values in bold indicate significant levels <0.05.

The total sample was 9,449, but 171 were missing for gender, so the total number of male and female students was 9,278. Bold indicates statistically significant.

### Comparison of academic performance of adolescents with meeting different types of physical activity guidelines

The Kruskal–Wallis test was used in this study to determine whether the levels of academic performance differed between the different types of adolescents who were met the guidelines. The normal quantile plot and the Shapiro–Wilk test suggested that in the four groups of NPA + NST, NPA + ST, PA + NST, and PA + ST, the adolescents’ scores in Chinese test, mathematic test, English test and school life experience did not follow a normal distribution. The Levene’s test suggested that the overall variance of the four groups was not homogeneous and qualified for the use of the Kruskal–Wallis test.

The results of the analysis showed that the Chinese test score levels of adolescents in the NPA + NST, NPA + ST, PA + NS, and PA + ST groups were, respectively, 47.24 (P_25_ ~ P_75_: 41.61 ~ 54.93), 48.52 (P_25_ ~ P_75_: 43.65 ~ 55.89), 48.40 (P_25_ ~ P_75_: 43.65 ~ 55.57), and 51.73 (P_25_ ~ P_75_: 48.06 ~ 55.22). The results of the Kruskal–Wallis test showed that the four groups did not all have the same level of Chinese test scores (*χ*^2^ = 31.234, *p* = 0.001). When analyzed by gender, among boys, the Chinese test score levels of adolescents in the NPA + NST, NPA + ST, PA + NST and PA + ST groups were, respectively, 49.73 (P_25_ ~ P_75_: 45.05 ~ 56.86), 49.67 (P_25_ ~ P_75_: 44.62 ~ 56.86), 50.97 (P_25_ ~ P_75_: 52.67 ~ 57.50) and 52.97 (P_25_ ~ P_75_: 52.67 ~ 57.50). The Kruskal–Wallis test showed that all four groups of boys had different levels of Chinese test scores (*χ*^2^ = 35.218, *p* = 0.001). Among girls, the Chinese test score levels of adolescents in the NPA + NST, NPA + ST, PA + NST and PA + ST groups were, respectively, 52.67 (P_25_ ~ P_75_: 48.48 ~ 58.15), 54.49 (P_25_ ~ P_75_: 51.71 ~ 58.36), 52.85 (P_25_ ~ P_75_: 49.13 ~ 58.15) and 55.37 (P_25_ ~ P_75_: 49.13 ~ 58.15). The results of the Kruskal–Wallis test showed that all four groups of girls had different levels of Chinese test scores (*χ*^2^ = 8.216, *p* = 0.042).

The results of the analysis showed that the levels of mathematics test scores of adolescents in the NPA + NST, NPA + ST, PA + NST, and PA + ST groups were, respectively, 49.59 (P_25_ ~ P_75_: 41.93 ~ 58.26), 50.14 (P_25_ ~ P_75_: 42.20 ~ 58.26), 51.38 (P_25_ ~ P_75_:44.87 ~ 58.90), and 53.84 (P_25_ ~ P_75_:44.87 ~ 58.90). The results of the Kruskal–Wallis test showed that the four groups of adolescents did not all have the same level of mathematics test scores (*χ*^2^ = 52.535, *p* = 0.001). When analyzed by gender, among boys, the mathematics achievement levels of boys in the NPA + NST, NPA + ST, PA + NST and PA + ST groups were, respectively, 48.48 (P_25_ ~ P_75_: 39.78 ~ 57.62), 49.39 (P_25_ ~ P_75_: 41.05 to 58.26), 50.30 (P_25_ ~ P_75_:42.64 ~ 58.64). The results of the Kruskal–Wallis test showed that the four groups of boys did not all have the same level of mathematics test scores (*χ*^2^ = 35.218, *p* = 0.001). Among the girls, the NPA + NST, NPA + ST, PA + NST and PA + ST groups of girls scored at 50.91 (P_25_ ~ P_75_: 44.36 ~ 58.70), 53.30 (P_25_ ~ P_75_: 50.99 ~ 58.264), 52.17 (P_25_ ~ P_75_: 46.47 ~ 58.90) and The results of the Kruskal–Wallis test showed that the four groups of girls did not all have the same level of mathematics test scores (*χ*^2^ = 14.705, *p* = 0.021).

The results of the analysis showed that the English test score levels of adolescents in the NPA + NST, NPA + ST, PA + NST and PA + ST groups were, respectively, 49.45 (P_25_ ~ P_75_:41.14 ~ 58.23), 50.83 (P_25_ ~ P_75_:42.85 ~ 58.98), 51.82 (P_25_ ~ P_75_: 45.44 ~ 59.77), and 53.41 (P_25_ ~ P_75_: 46.67 ~ 61.65). The results of the Kruskal–Wallis test showed that the four groups did not all have the same level of English test scores (*χ*^2^ = 98.081, *p* = 0.001). When analyzed by gender, in boys, the English test score levels of adolescents in the NPA + NST, NPA + ST, PA + NST, and PA + ST groups were, respectively, 46.90 (P_25_ ~ P_75_: 37.85 ~ 55.84), 49.72 (P_25_ ~ P_75_: 41.55 ~ 58.16), 49.55 (P_25_ ~ P_75_: 41.65 ~ 58.23), and 51.70 (P_25_ ~ P_75_:45.24 ~ 60.42) Kruskal–Wallis tests showed that the four groups of adolescents did not all have the same level of English test scores (*χ*^2^ = 69.963, *p* = 0.001). Among girls, the English test score levels of girls in the NPA + NST, NPA + ST, PA + NST and PA + ST groups were, respectively, 51.46 (P_25_ ~ P_75_:46.74 ~ 59.60), 55.49 (P_25_ ~ P_75_:52.62 ~ 61.24), 53.47 (P_25_ ~ P_75_:48.32 ~ 60.42) and The results of the Kruskal–Wallis test showed that the four groups of girls did not all have the same level of English test scores (*χ*^2^ = 28.327, *p* = 0.001).

The results of the analysis showed that the school life experience levels of adolescents in the NPA + NST, NPA + ST, PA + NST, and PA + ST groups were, respectively, 23.67 (P_25_ ~ P_75_:21 ~ 26),25.60 (P_25_ ~ P_75_:23 ~ 28),23.94 (P_25_ ~ P_75_:21 ~ 24) and The Kruskal–Wallis test showed that the four groups did not all have the same level of school life experience (*χ*^2^ = 100.885, *p* = 0.001). When analyzed by gender, in boys, the school life experience levels of boys in the NPA + NST, NPA + ST, PA + NST, and PA + ST groups were, respectively, 23.81 (P_25_ ~ P_75_:21 ~ 27), 25.59 (P_25_ ~ P_75_:23 ~ 28), 24.04 (P_25_ ~ P_75_: 21 ~ 27), and 25.31 (P_25_ ~ P_75_:21 ~ 27). The Kruskal–Wallis test showed that the four groups of boys did not all have the same level of school life experience (*χ*^2^ = 56.064, *p* = 0.001). In girls, the levels of school life experience for girls in the NPA + NST, NPA + ST, PA + NST, and PA + ST groups were, respectively, 23.51 (P_25_ ~ P_75_: 21 ~ 26), 25.66 (P_25_ ~ P_75_:23 ~ 28), 23.86 (P_25_ ~ P_75_:22 ~ 26), and 26.02 (P_25_ ~ P7_5_:24 ~ 27). The results of the Kruskal-Wallis test showed that the four groups of girls did not all have the same level of school life experience (*χ*^2^ = 41.826, *p* = 0.001).

**Figure 1:**
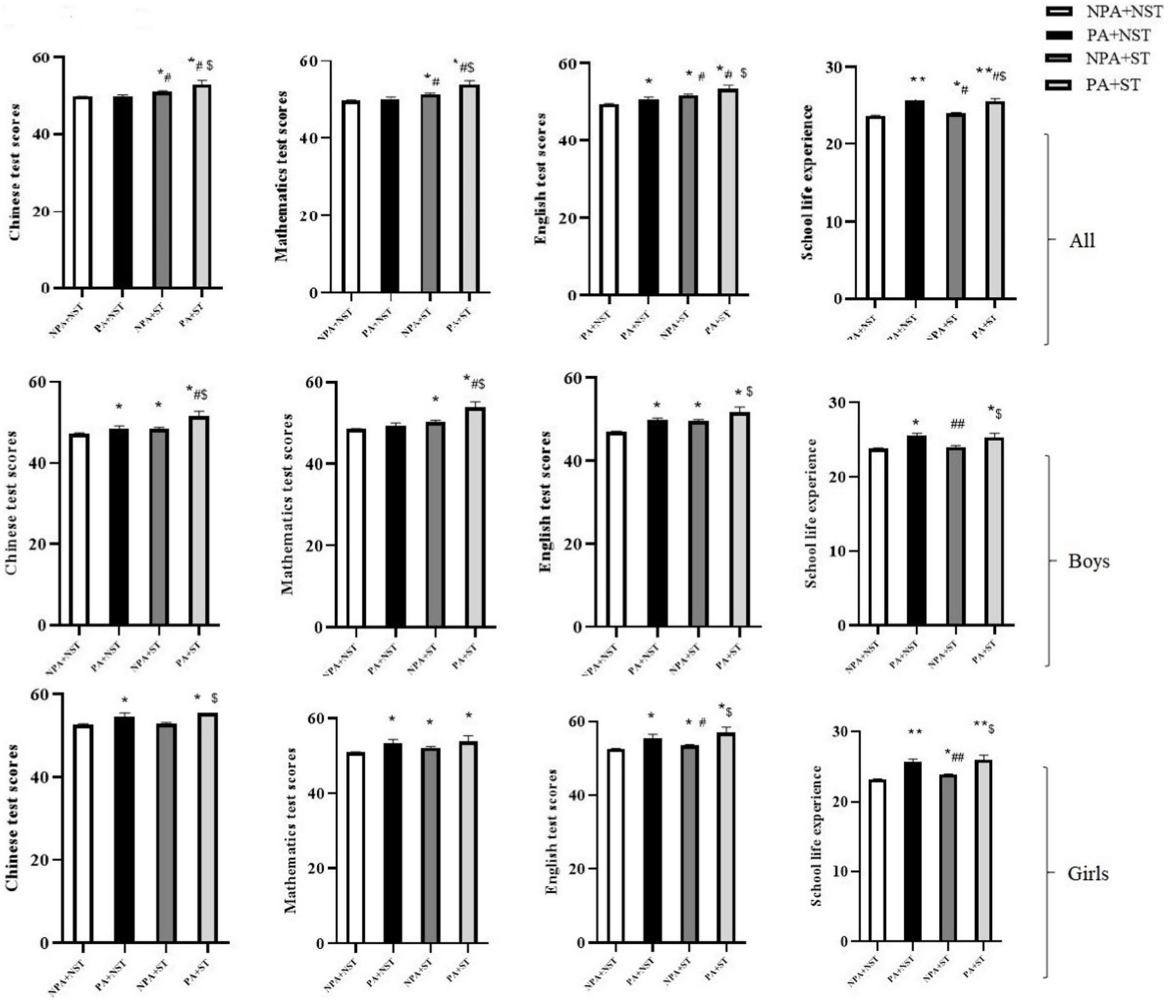
Academic performance of adolescents in different categories.* represents comparison with the NPA + NST group, *p* < 0.05; ** represents comparison with the NPA + NST group, *p* < 0.01; # represents comparison with the PA + NST group, *p*  < 0.05; ## represents comparison with the PA + NST group, *p* < 0.01. $ represents comparison with NPA + ST group, *p* < 0.05.

The comparison of the Chinese test scores, mathematics test scores, English test scores and school life experience scores of the different groups of young people is shown in Figure 1.

### The relationship between meeting physical exercise time and screen time and adolescent academic performance

Adolescent academic performance may be influenced by covariates and multiple regression was used to better estimate the relationship between meeting guideline recommendations and adolescent academic performance. Multiple linear regression models were used to analyze the relationship between meeting the different recommendations of the guidelines and adolescents’ academic performance (Table 2). After adjusting for covariates (e.g., age, gender, ethnicity, etc.), adolescents in the PA + NST group had a 1.00 unit increase in English test scores (*t* = 2.16, *p* = 0.031) and a 1.72 unit increase in School life experience (*t* = 7.40, *p* < 0.001). Compared to the reference group of adolescents in the NPA + NST group, adolescents in the NPA + ST group had a 0.97 unit increase in mathematics test scores (*t* = 3.89, *p* < 0.001), a 1.03 unit increase in English test scores (*t* = 4.35, *p* < 0.001), and a 0.27 unit increase in school-life experience (*t* = 2.38, *p* = 0.018). Adolescents in the PA + ST group had a 2.74 unit increase in mathematics test scores (*t* = 2.94, *p* = 0.003), a 2.39 unit increase in English test scores (*t* = 2.74, *p* = 0.006), a 2.31 unit increase in Chinese test scores (*t* = 2.57, *p* = 0.010), and a 1.72 unit increase in School-life experience increased by 1.72 units (*t* = 3.98, *p* < 0.001). Overall, meeting both physical activity and screen time requirements had a more significant impact on adolescent test scores in mathematics, Chinese test scores, English test scores and School life experience.

**Table 2:** The relationship between meeting physical exercise time and screen time and adolescent academic performance.

	Math	English	Chinese	School-life experience
	Coef. (95%CI)			
PA + NST
All	−0.25	1.00*	0.72	1.72**
	(−1.22 ~ 0.73)	(0.09 ~ 1.92)	(−0.87 ~ 1.02)	(1.26 ~ 2.18)
Boys	−0.61	0.91	−0.20	1.70**
	(−1.76 ~ 0.53)	(−0.18 ~ 2.01)	(−1.40 ~ 0.98)	(1.14 ~ 2.25)
Girls	1.13	1.45	0.99	1.85**
	(−0.93 ~ 3.20)	(−0.43 ~ 3.34)	(−0.78 ~ 2.76)	(0.91 ~ 2.79)
NPA + ST
All	0.97**	1.03**	0.22	0.27*
	(0.48 ~ 1.47)	(0.45 ~ 1.30)	(−0.25 ~ 0.70)	(0.04 ~ 0.51)
Boys	1.15**	1.79**	0.59	0.24
	(0.37 ~ 1.94)	(1.04 ~ 2.54)	(−0.21 ~ 1.42)	(−0.13 ~ 0.62)
Girls	0.83**	0.41	−0.50	0.31**
	(0.20 ~ 1.45)	(−0.15 ~ 0.98)	(−0.60 ~ 10.46)	(0.03 ~ 0.59)
PA + ST
All	2.74**	2.39**	2.31**	1.72**
	(0.91 ~ 4.57)	(−0.09 ~ 3.05)	(0.54 ~ 4.08)	(0.87 ~ 2.57)
Boys	3.42**	1.46*	2.59**	1.46**
	(1.07 ~ 5.77)	(0.04 ~ 4.53)	(0.14 ~ 5.03)	(0.33 ~ 2.58)
Girls	1.15	2.42	1.49	2.32**
	(−1.82 ~ 4.13)	(−0.30 ~ 5.15)	(−1.06 ~ 4.05)	(0.99 ~ 3.64)

*represents *p* < 0.05 compared with the reference group; **represents *p* < 0.01 compared with the reference group.

When analyzed by gender, in boys, after adjusting for covariates (e.g., age, gender, ethnicity, etc.), the School-life experience of boys in the PA + NST group increased by 1.70 units (*t* = 6.07, *p* < 0.001) compared to boys in the reference NPA + NST group. Boys in the NPA + ST group had a 1.79 unit increase in English test scores (*t* = 4.69, *p* < 0.001) and a 1.15 unit increase in mathematics test scores (*t* = 2.90, *p* = 0.004) compared to boys in the reference NPA + NST group. Compared to the reference group of boys in the NPA + NST group, the Boys in the PA + ST group had a 3.42 unit increase in mathematics test scores (*t* = 2.86, *p* = 0.004), a 1.46 unit increase in English test scores (*t* = 2.08, *p* = 0.038), a 2.59 unit increase in Chinese test scores (*t* = 2.00, *p* = 0.046), and a 1.46 unit increase in School life experience (*t* = 2.55,*p* = 0.011). Overall, meeting both physical exercise and screen time requirements had a more significant impact on boys’ mathematics test scores, Chinese test scores and School life experience.

In girls, after adjusting for covariates (e.g., age, gender, ethnicity, etc.), the School-life experience of girls in the PA + NST group increased by 1.85 units (*t* = 3.90, *p* < 0.001) compared to girls in the reference NPA + NST group. Girls in the NPA + NST group had a 0.83 unit increase in mathematics test scores (*t* = 2.62, *p* = 0.009) and a 0.31 unit increase in school-life experience (*t* = 2.20, *p* = 0.028) compared to girls in the reference NPA + NST group. School life experience increased by 2.32 units for girls in the PA + ST group compared to girls in the reference group NPA + NST (*t* = 3.43, *p* = 0.001). Overall, the impact of meeting both physical exercise time and screen time requirements was more significant for girls’ School life experience.

## Discussion

To our knowledge, this was the first study to examine the relationship between the Physical Activity Guidelines for Chinese Residents and the academic performance of Chinese adolescents. The results of this study showed that meeting the physical exercise time and screen time in the Physical Activity Guidelines for Chinese Residents was associated with adolescents’ academic performance. Specifically, having at least 60 min of physical exercise per day was associated with adolescents’ school life experiences compared to adolescents who did not meet the physical exercise time and screen time in the Physical Activity Guidelines for Chinese Residents. Several previous studies have reported that students with more physical exercise time have higher academic potential (Lindner, 2002; Augestad and Jiang, 2015). Mitchell et al. (2015) found that increasing adolescent physical exercise time improved adolescents’ autonomy, competence and basic psychological needs related to their environment (Mitchell et al., 2015). Cumulative screen time of less than 2 h per day was associated with adolescents’ mathematics test scores, English test scores and school life experiences compared to adolescents who did not meet the physical exercise and screen time guidelines of the Chinese Resident Physical Activity Guidelines. This was consistent with the findings of Trinh et al. (2015) and Yan et al. (2017). Meeting both physical exercise time and screen time recommendations had more significant effects on adolescents’ mathematics, Chinese, English, and school life experiences. Notably, there were some differences between boys and girls. Meeting both the physical exercise time and screen time recommendations in the Chinese Residents’ Physical Activity Guidelines had more significant effects on boys’ Maths test scores, Chinese test scores, and School life experience. In contrast, meeting both the physical exercise time and screen time recommendations in the Physical Activity Guidelines for Chinese Residents only had a more significant effect on School life experience for girls.

Although the results of this study support the positive impact of adolescents meeting the recommendations of the Physical Activity Guidelines for Chinese Residents (2021) in terms of physical exercise time and screen time on adolescents’ academic performance. However, it was worth noting that only 3.81% of the adolescents in this study achieved the minimum of 60 min of physical exercise per day as recommended in the Physical Activity Guidelines for Chinese Residents. 17.62% of adolescents in the sample accumulated less than 2 h of screen time per day. 1.01% of adolescents participated in physical exercise for at least 60 min per day and accumulated less than 2 h of screen time per day. This implies that, based on the results of the current study, Chinese adolescents often fail to comply with the recommendations for physical exercise time and screen time for adolescents in the Chinese Resident’s Guide to Physical Activity and may be a common problem among the adolescent population. However, as the present study has confirmed that adolescents’ adherence to the Physical Activity Guidelines for Chinese Residents’ physical exercise time and screen time can predict adolescent academic performance, researchers in the field should continue to investigate how expanding adherence to the Physical Activity Guidelines for Chinese Residents’ physical exercise time and screen time recommendations for adolescents would benefit academic performance in different real-life scenarios, and the practical difficulties young people face in meeting physical activity guidelines. Schools, communities and others should create more physical exercise environments for groups of adolescents, and families should exercise more supervision over the use of screen equipment by groups of adolescents. Encourage more active physical activity behaviors in adolescent groups and policy makers should further work toward promoting physical activity well-being in adolescent groups.

The present study has a number of important strengths, including a large sample size, adjustment for a variety of confounding factors, and a comprehensive description of academic performance that combines objectively measured core subject test scores with self-reported student life experiences. And stratified analyses using gender to explore differences in the impact of physical exercise time and screen time on the academic performance of adolescents by gender are also valuable. In addition, this study provides insight into the factors that influence adolescent academic performance in a Chinese social context, and these findings may promote greater emphasis on physical activity and reduced screen time for adolescents in schools and families.

This study also has some limitations, which should be taken into account when interpreting the results of this study. Physical activity status and screen time were self-reported by adolescents and therefore may have encountered the effects of misreporting, which may have weakened the associations observed in the study. Although, adolescent physical activity guideline attainment based on these self-reports may be underestimated, this self-report has been widely used in demographic studies and its values are closely related to adolescent physical activity values measured in most cases. Second, although some potential moderators of the association between physical activity and academic performance have been documented in the literature (e.g., self-efficacy), the present study was unable to examine these mediating effects in the study because they were not available in the original data obtained for this study. Third, this study could not control for some potential confounders (e.g., physical health) because these data were also not available. Fourth, the data used in this study are cross-sectional and can only provide an overview of current physical exercise and screen time, and cannot indicate how past experiences of physical exercise time and screen time affect adolescents’ current academic performance. That is, it is not possible to answer whether long-term physical exercise and screen time have a cumulative effect on academic performance.

## Conclusion

Participation in physical exercise for at least 60 min per day and/or less than 2 h of cumulative screen time per day were both associated with adolescent academic performance.

## Data availability statement

Publicly available datasets were analyzed in this study. This data can be found at: http://ceps.ruc.edu.cn/.

## Ethics statement

The public dataset from the official CEPS website (http://ceps.ruc.edu.cn/) was used for this study. Therefore, the Academic Committee of Shandong Institute of Physical Education waived the requirement for ethical approval. CEPS provided respondents with guarantees of privacy and confidentiality. Written informed consent was provided by all participants. Detailed information about the study design can be found on their official website.

## Author contributions

LL conceived the research, and performed data analysis and interpretation. JY and HW drafted the manuscript. LL is mainly responsible for the revision of the thesis. All authors have read and approved the final manuscript.

## Funding

Funding for this research came from the Guizhou Provincial Department of Education Youth Growth Project Fund (Qianjiao He KY (2021) 291), and the Guizhou Province Education Planning Fund Project (2021A058).

## Conflict of interest

The authors declare that the research was conducted in the absence of any commercial or financial relationships that could be construed as a potential conflict of interest.

## Publisher’s note

All claims expressed in this article are solely those of the authors and do not necessarily represent those of their affiliated organizations, or those of the publisher, the editors and the reviewers. Any product that may be evaluated in this article, or claim that may be made by its manufacturer, is not guaranteed or endorsed by the publisher.

## Supplementary material

The Supplementary material for this article can be found online at: https://www.frontiersin.org/articles/10.3389/fpsyg.2023.1002839/full#supplementary-material
