# Supplementary material for: The association between meeting physical activity guidelines and academic performance among junior high school adolescents in China-evidence from the China education tracking survey
Source: Front Psychol. 2023 Feb 16;14:1002839. doi: 10.3389/fpsyg.2023.1002839 (PMC9978766; doi:10.3389/fpsyg.2023.1002839)
Supplement: Supplementary file 1 [file Table_1.DOCX]

**Supplementary file 1：**

Please read each item carefully to understand its meaning, and then choose the answer that best suits you according to your actual situation, and tick the corresponding number with "√".Do you agree with the following statements about school life.

(1.Completely disagreet;2.Disagree more;3.Agree more;4.Completely agree )

| 1.My parents often received praise from my teachers | 1□ | 2□ | 3□ | 4□ |
| --- | --- | --- | --- | --- |
| 2.My parents often received criticism from my teachers | 1□ | 2□ | 3□ | 4□ |
| 3.My class teacher always praises me | 1□ | 2□ | 3□ | 4□ |
| 4. My class teacher often criticises me | 1□ | 2□ | 3□ | 4□ |
| 5. Most of the students in the class were friendly to me | 1□ | 2□ | 3□ | 4□ |
| 6.I am in a class with a good culture | 1□ | 2□ | 3□ | 4□ |
| 7.I often take part in school or classroom organised activities | 1□ | 2□ | 3□ | 4□ |
| 8.I feel close to the people of this school | 1□ | 2□ | 3□ | 4□ |
| 9.I am bored in this school | 1□ | 2□ | 3□ | 4□ |
| 10.I wish I could go to another school | 1□ | 2□ | 3□ | 4□ |
